# Differential Effects of Motor Efference Copies and Proprioceptive Information on Response Evaluation Processes

**DOI:** 10.1371/journal.pone.0062335

**Published:** 2013-04-26

**Authors:** Ann-Kathrin Stock, Edmund Wascher, Christian Beste

**Affiliations:** 1 Institute for Cognitive Neuroscience, Department of Biopsychology, Ruhr-UniversityBochum, Bochum, Germany; 2 Leibnitz-Institut für Arbeitsforschung an der TU Dortmund, Abt. ahrnehmungskybernetik, Dortmund, Germany; University of Ottawa, Canada

## Abstract

It is well-kown that sensory information influences the way we execute motor responses. However, less is known about if and how sensory and motor information are integrated in the subsequent process of response evaluation. We used a modified Simon Task to investigate how these streams of information are integrated in response evaluation processes, applying an in-depth neurophysiological analysis of event-related potentials (ERPs), time-frequency decomposition and sLORETA. The results show that response evaluation processes are differentially modulated by afferent proprioceptive information and efference copies. While the influence of proprioceptive information is mediated via oscillations in different frequency bands, efference copy based information about the motor execution is specifically mediated via oscillations in the theta frequency band. Stages of visual perception and attention were not modulated by the interaction of proprioception and motor efference copies. Brain areas modulated by the interactive effects of proprioceptive and efference copy based information included the middle frontal gyrus and the supplementary motor area (SMA), suggesting that these areas integrate sensory information for the purpose of response evaluation. The results show how motor response evaluation processes are modulated by information about both the execution and the location of a response.

## Introduction

Being able to monitor and evaluate our movements and actions is essential to human behavior. It allows us to perform complex and precise operations, correct for errors, and quickly adapt to unexpected changes in our environment [1]–[2]. These processes can be fairly complex since the representation of most of our actions and goals comprises several aspects. For example, a motor response often needs to get exerted in the right place in order to have the desired effect. Hence, defining response evaluation as the integration of information relevant to the desired response involves that response evaluation processes need to comprise detailed information on both the motor execution and the location of a given response [3]. However, this information is usually obtained via different sources:

Movements, especially those of limbs, are planned and executed with the help of cortical networks comprising the supplementary motor area (SMA) and area M1 [4], [5]. Internal copies of efferent motor signals sent to our limbs are retained in the respective brain regions for further processing, especially in the SMA [5]–[9]. Motor efference copies have been shown to be involved in the processing of errors [10] as well as a wide range of other processes like motor control and execution [11]–[17] visual perception [10], [18]–[20], posture [21], auditory [22] and tactile perception [16].

Afferent sensory feedback obtained from the peripheral effectors of our body provides information on their position as well as the results of a response and the necessity of adaptations. It has been shown that purely afferent information on changes in proprioception does influence EEG measures [23], [24] including error monitoring [11]. Just like for motor commands, findings indicate that different aspects of spatial (egocentric) sensory information are represented in the SMA [25].

Taken together, information on the motor execution of a response (efference motor copies) and its location (afferent proprioceptive input) are based on at least partly different and independent neuronal networks. Yet, both proprioceptive and motor information are processed within the SMA [5]–[7], [25]. Therefore, the question of if and how they are integrated within the SMA is pivotal. Even though there are currently no studies answering this question with respect to response evaluation, there are many studies of spatial attention dealing with this issue.

For spatial attention, it is assumed that there are cross-modal links between vision, audition, and touch (e.g. [26]). In this context, multimodal neurons integrating visual and postural information have been found in the monkey homologue of premotor areas (area 6, see [27]–[28]). Yet still, these findings do not explain according to which principles afferent and efferent information are accounted for within SMA.

Motor responses are mainly generated in the hemisphere contralateral to the involved hand (e.g. [5]) and the associated efference motor copies are usually retained in the hemisphere in which they were generated [7].

Sensory input (including proprioceptive information) is initially represented modality-specific and spatially mapped (retinotopic, tonotopic, somatotopic). In a fixed posture (parallel hands, straight gaze), sensory stimuli project to the respectively contralateral hemisphere [26]. Since most experimental paradigms with manual responses are conducted with a parallel hands posture, the results can yield information about spatial processing, but these findings cannot be interpreted with respect to the question of whether the required spatial information is embedded in an egocentric (somatotopic, hemisphere-based) or an external reference system. Until recently, studies of healthy and impaired subjects hence led to the conclusion that sensorimotor and somatosensory spatial representations are rather somatotopic (e.g. [29]). However, the fact that the some body parts including the sensory organs can move quite independently poses the challenge of transforming and/or integrating the incoming afferent sensory information so that a proper interaction with the stimuli of the environment can be maintained at any posture [26]. While some aspects of spatial attention seem to be confined to the hemispheres of the brain [25], [30], there is now converging evidence that this does not hold true for all aspects of spatial information processing: When it comes to cross-modal attention, initial hemispheric projections seem be overcome so that sensory events can be mapped to “common external locations” or external reference frames [26], [30]–[35]. There is even support for the assumption that sensory stimuli are remapped into external spatial coordinates irrespective of any task requirements [36]–[37]. The benefit of such remapping is self-evident since “the use of both anatomical and external coordinates may facilitate the control of actions toward tactile events and the choice of the most suitable effector.” [36].

Based on these findings, we hypothesize that response monitoring processes might resemble the mechanisms of spatial attention. We therefore expect that:

Efference motor copies are at least partly retained in the hemisphere contralateral to the anatomical site of the responding hand.

Sensory (proprioceptive) information is remapped into external spatial coordinates. As a consequence, its assignment to the hemispheres of the brain should depend rather on external than on internal spatial coordinates.

Allocating efferent and afferent pieces of information in different hemispheres might exacerbate their integration, possibly resulting in a processing conflict and/or difficulties to integrate this information across hemispheres.

The question of whether cross-modal information integration is based on initial hemispheric projections or on external spatial coordinates can be assessed with the help of a task in which hand positions are varied in space. -Only in the case of an external reference frame, crossing hands should influence or even reverse behavioral and electrophysiological measures [33]. As Eimer et al. [26] put it “crossing the hands results in a conflict within somatosensory information-processing, between a spatial code referring to the position in external space where the hand is currently located, and a spatial code representing the anatomical side of the responding hand.” Hence, we used a modified version of the Simon Task [38]–[39]. It allows for a dissociation of efference (motor) copies and afferent postural information (proprioception) by making the subjects respond with different hands and by altering the spatial position of the responding hands (see methods section for further details). To obtain response monitoring-related measures, we integrated behavioral data, event-related potentials (ERPs), time-frequency (TF) decomposition and source localization methods (sLORETA). As explained above, response evaluation processes of both correct and erroneous reactions can be measured via fronto-central ERPs, which most likely reflect activation of the SMA (as well as the anterior cingulate cortex and adjacent areas) [15], [40]–[49]. Also, changes in spatial attention and posture have been shown to influence the negativity at midline electrodes [26], [50] and SMA activity [5]. According to our hypotheses we therefore expected to find that changes in proprioceptive feedback alter the distribution of SMA-dependent response monitoring processes between the hemispheres as measured via fronto-central EEG potentials and source localization. Considering that frequency bands can probably be differentially modulated by cognitive (sub)processes and that theta as well as delta oscillations have previously been linked to response monitoring processes [17], [47], [51]–[52], we mainly focused on the delta and theta frequency bands.

## Materials and Methods

### 2.0 Ethics Statement

All subjects gave written consent and were treated in accordance with the declaration of Helsinki. The study was approved by the ethics committee of the medical faculty of the University of Bochum.

### 2.1 Sample

This study’s sample consists of 25 healthy right-handed volunteers (13 male, 12 female). Handedness was assessed using the Edinburgh Handedness Inventory [53]; the mean EHI score was 0.90 (SD = 0.14, range from 0.5 to 1). The mean age was 23.17 years (SD = 2.37, range from 20 to 29). None of the subjects presented with a history of psychiatric or neurological disease as reported in a customized questionnaire designed by experienced neuropsychologists. Each subject received a reimbursement of 10 € after the participation in the experiment was completed.

### 2.2 Setting and Task

**Figure 1:**
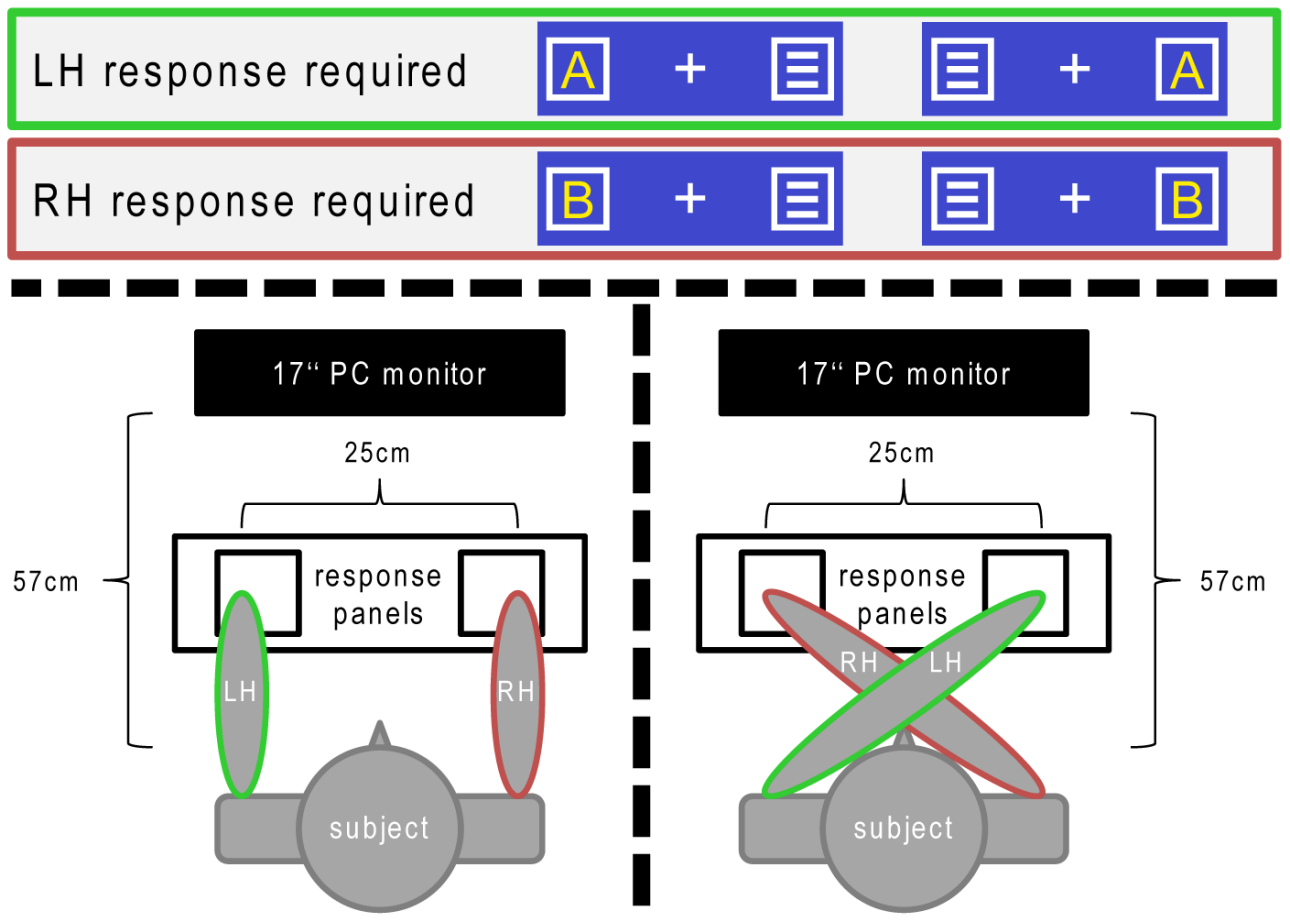
Illustration of the experimental setting. The target stimuli (letters) could be located in either of the boxes as illustrated in the top rows. Letter A required a reaction of the left hand (respective box and limbs edged green) while letter B required a reaction of the right hand (respective box and limbs edged red). The parallel hands condition is depicted in the bottom left part of the figure while the crossed hand condition is depicted on the right side.

The experimental setup is depicted in Fig. 1. All subjects were comfortably seated at a distance of 57 cm from a 17 inch CRT computer monitor in a dimly lit and sound-attenuated room. The distance to the screen was controlled using a custom-made head support mounted on the table carrying the monitor. Responses were recorded using two buttons located on two different custom-made response panels placed in front of the subjects. The panels were attached to a board so that they were fixed at horizontal distance of 11 cm (inner boundaries), resulting in a horizontal distance of 25 cm between the two buttons. For the presentation of stimuli as well as for the recording of the responses (reaction times (RTs) and correctness), Presentation (version 14.9. by Neurobehavioral Systems, Inc.) was used. The design of the Simon Task used in this study references that used by Wascher et al. [39], yet some modifications had been made. The background color was set to dark blue and a white fixation cross was continuously displayed in the center of the screen. Also, two white frame boxes were laterally presented at the same vertical level as the fixation cross. The distance of the inner border of the two lateral boxes from the fixation cross was 1.1°. Like the fixation cross, the two boxes remained on the screen throughout the experiment. Each trial began with the presentation of a yellow capital letter (A or B) as a target stimulus within one of the boxes while the other box contained a noise stimulus (three horizontal bars). Stimuli were approximately 0.5° wide and 0.6° high. Both target and noise stimuli were presented simultaneously for 200 ms, after which the empty boxes remained on the screen. The first given response (button press) ended the trial. In cases in which the response did not occur within the first 500 ms after the onset of the trial, a speed-up sign (containing the German word “Schneller!” which translates to “Faster!”) was presented above the stimuli until the end of the trial. If no response was given, the trial automatically ended 1700 ms after its onset and was coded as a “miss”. The trials were separated by response-stimulus intervals (RSIs) during which the fixation cross and the two boxed remained on the screen. The duration of the RSIs varied randomly and ranged between 2000 and 2500 ms. The experiment was made up of eight blocks, each consisting of 100 trials. All four conditions (as defined via the spatial S-R correspondence/correspondence of stimulus and response site as well as via the positioning of hands) occurred equally often, resulting in 25 trials per condition and block. The order of the trials was pseudorandomized. Each block was preceded by a detailed instruction presented on the screen and followed by a pause the length of which could be determined by the subjects. For half of the blocks (blocks 1, 3, 5, and 7) the subjects were instructed to place their arms onto the response panels in parallel so that the left index finger was located on the left response button and the right index finger was located on the right response button. For the remaining four blocks (blocks 2, 4, 6, and 8) the subjects were instructed to cross their arms (with the left arm being on top of the right arm) so that the left index finger was placed on the right response button and vice versa. Given that the data was analyzed using a within-subject design, we do not expect the uniform order of hand postures in all subjects to systematically bias the results. In all blocks (uncrossed and crossed), the subjects were instructed to use their left index finger to respond to the letter A target stimulus (irrespective of its location on the screen) while the right index finger should be used to respond to the letter B target stimulus in an analogue fashion (see Fig. 1 for illustration). All trials in which the target stimulus and the correct response button were located in the same hemifield were classified as spatially correspondent. Hence, all trials in which the stimulus and the button were located in opposing hemifields were classified as spatially non-correspondent.

### 2.3 Behavioral Data Processing

For each subject, mean RTs and error rates were extracted for the four conditions (correspondent & parallel hands/non-correspondent & parallel hands/correspondent & crossed hands/non-correspondent & crossed hands). In addition to the calculations described below, a vincentizing procedure was applied to the individual response time data [54]–[55]. The results of the vincentizing procedure are shown in the supporting information (Text S1).

### 2.4 EEG Data Recording and Processing

While the participants were performing the Simon Task, an EEG was recorded from 65 Ag–AgCl electrodes at standard positions (international 10–20 system). Electrode FCz was used as the primary reference. Applying a filter bandwidth of 0–80 Hz, EEG data was recorded with a sampling rate of 1000 samples per second. Electrode impedances were kept below 5 kΩ. IIR filtering was applied offline in the band-pass from 0.5 to 18 Hz (using a slope of 48 dB/oct). Data sets were visually inspected and all segments contaminated by technical artifacts were rejected. An independent component analysis (ICA) applying the infomax algorithm was used to remove horizontal and vertical eye movements, as well as pulse artifacts from the unepoched data sets. For the analysis of response-locked event-related potentials (ERPs), segments were formed for the different conditions. Epochs started 1200 ms before the response (which was set to time point zero) and ended 1200 ms after the response, resulting in an epoch length of 2400 ms. Only trials that had been correctly answered within the first 1500 ms after the onset of the stimulus presentation were included. An automated artifact rejection procedure was run using a maximum voltage step of more than 50 µV/ms, a maximal value difference of 200 µV in a 200 ms interval, or activity below 0.5 µV as rejection criteria. To re-reference the data, a current source density (CSD) transformation was applied. The CSD transformation eliminates the reference potential and works as a spatial filter that enhances differences between the electrodes, thus reducing spatial dispersion of neuronal signals [56]–[59]. As a consequence, the ERPs are more confined to the relevant electrodes in the topographic center of activity. The resulting CSD values are given in µV/m^2^ so that amplitude values derived therefrom cannot be directly compared to data that has not undergone a CSD transformation. For the time-domain analysis, a baseline correction was referred to −1200 ms till −800 ms (before the response) to eliminate background activity. The epochs of the different conditions were then averaged. Based on scalp topography maps (see results section/response-locked ERPs and scalp topographies), the physical properties of the averaged potentials (including the signal-to-noise-ratio) and based on studies linking FC electrodes to SMA activity [60], we selected electrodes FC1 and FC2 (formerly C3’ and C4’; cf. [61]) for subsequent in-depth analyses of the negative fronto-central potentials most likely associated with response evaluation processes. Consequently, a semiautomatic peak detection was used to identify local maxima (between-150 ms and 0 ms) and minima (between 0 ms and 150 ms) for electrodes FC1 and FC2. The time frames were chosen in order to quantify the first post-response peak (which according to the grand average of all subjects was negative). The last (positive) pre-response peak was classified in the same manner in order to be able to form peak-to-peak values in addition to the regular peak-to-baseline values. Hence, peak-to-peak values were formed by subtracting the values of the local minima from those of the local maxima on an individual level. The program BrainVision Analyzer (version 2.0.1.5528) was used for both EEG data preprocessing and the time-frequency decomposition described below.

In addition to the response-locked data, stimulus-locked potentials were formed for in order to validate the obtained data and to compare it to the findings of previous studies [61]. Since these parts of our calculations are not relevant to the exploration of our hypothesis, they can be found in the supporting information (Text S2).

### 2.5 Time-frequency Decomposition (Response-locked Wavelets)

Only trials in which a correct response occurred within 1500 ms after the onset of the respective stimulus were included in the time-frequency (TF) decomposition. Also, the trials were subdivided into sixteen conditions as defined by spatial S-R correspondence, hand position, used hand and motor execution (In this context, we would like use the term “motor execution” to describe whether the respective electrode was located above the motor cortex of the hemisphere in charge of the motor execution of the response). The proceeding of the TF decomposition was mainly based on the methodological descriptions by Ocklenburg et al. [62]. The epochs used for TF decomposition were set to start 2000 ms before and end 2000 ms after the response and were based on the CSD-transformed data. The time point of the response was set to zero. The resulting epoch length of 4000 ms allows for a reliable measurement of slow oscillations as defined for the delta and theta frequency bands [62]. In a first step, the epochs underwent an artifact rejection (same criteria as described above), a CSD transformation and a baseline correction (from −1200 ms to −800 ms) in order to estimate the background activity. Subsequently, TF analyses were performed for averaged response-related potentials (RRPs) to obtain evoked wavelet power. After this, the convolution with the complex wavelet was performed on this RRP average [63]. This was done separately for all of the sixteen different abovementioned conditions as follows: The TF analysis of the potentials was implemented using a continuous wavelet transform (CWT) based on Morlet Complex wavelets. The TF energy on the response was analyzed employing a modification of an already previously described method [64]. With the following equation, Complex Morlet wavelets *w* can be generated in the time domain for different frequencies *f*:
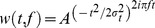
Where *t* is time, 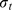
is the wavelet duration, 
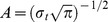
and 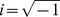.

For analysis and TF plots, a ratio of 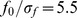 was used, with 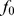 being the central frequency and 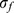 being the width of the Gaussian shape in the frequency domain. For different values of 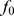, time and frequency resolutions can be calculated as 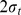 and 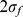, since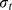 and 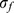 are related by the equation 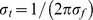 [65]. The analysis was performed in a frequency range of 0.5–18 Hz with a central frequency at 0.5 Hz intervals in 54 logarithmic steps. For different 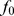
_,_ time and frequency resolutions (or wavelet duration and spectral bandwidth; [64]) can be calculated as 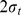 and 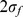 respectively. 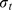 and 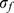 are related by the equation 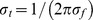. For example, for 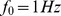, 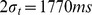 and 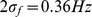; for 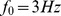, 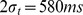 and 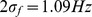; for 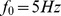, 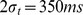 and 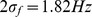. For the evoked wavelet power quantification, the data was normalized to the power of the baseline period. In order to obtain normal distribution of the TF power values for the subsequent statistical analysis, all values were log10-transformed as described by Beste et al. [65]–[66]. Finally, relevant TF components were extracted based on the observed data patterns (compare results section 3.2.2, response-locked ERPs and scalp topographies). This approach resulted in the quantification of the evoked power of the delta frequency band (at 2.07 Hz) and the theta frequency band (at 5.7 Hz) in electrodes FC1 and FC2. These electrodes were chosen on the basis of the results of the time-domain analysis (see results section).

### 2.6 sLORETA

Source localisation was conducted using sLORETA (standardized low resolution brain electromagnetic tomography [67]). sLORETA gives a single linear solution to the inverse problem based on extra-cranial measurements with no localization bias [67]–[68]. For sLORETA, the intracerebral volume is partitioned in 6239 voxels at 5 mm spatial resolution and the standardised current density at each voxel is then calculated in a realistic head model [69] using the MNI152 template [70]. Based on the results from response-locked ERP decomposition analyses, the voxel-based sLORETA-images were compared between the conditions of parallel and crossed hands (for every combination of used hand and S-R correspondence individually) using the sLORETA-built-in voxel-wise randomisation tests with 3000 permutations based on statistical non-parametric mapping. Voxels with significant differences (*p*<.05, corrected for multiple comparisons) between contrasted conditions were located in the MNI-brain and Brodman areas (BAs) as well as coordinates in the MNI-brain and were determined using the software (www.unizh.ch/keyinst/NewLORETA/sLORETA/sLORETA.htm). The comparison of sLORETA images between conditions was based on the response-locked ERPs.

### 2.7 Statistical Analysis

**Figure 2:**
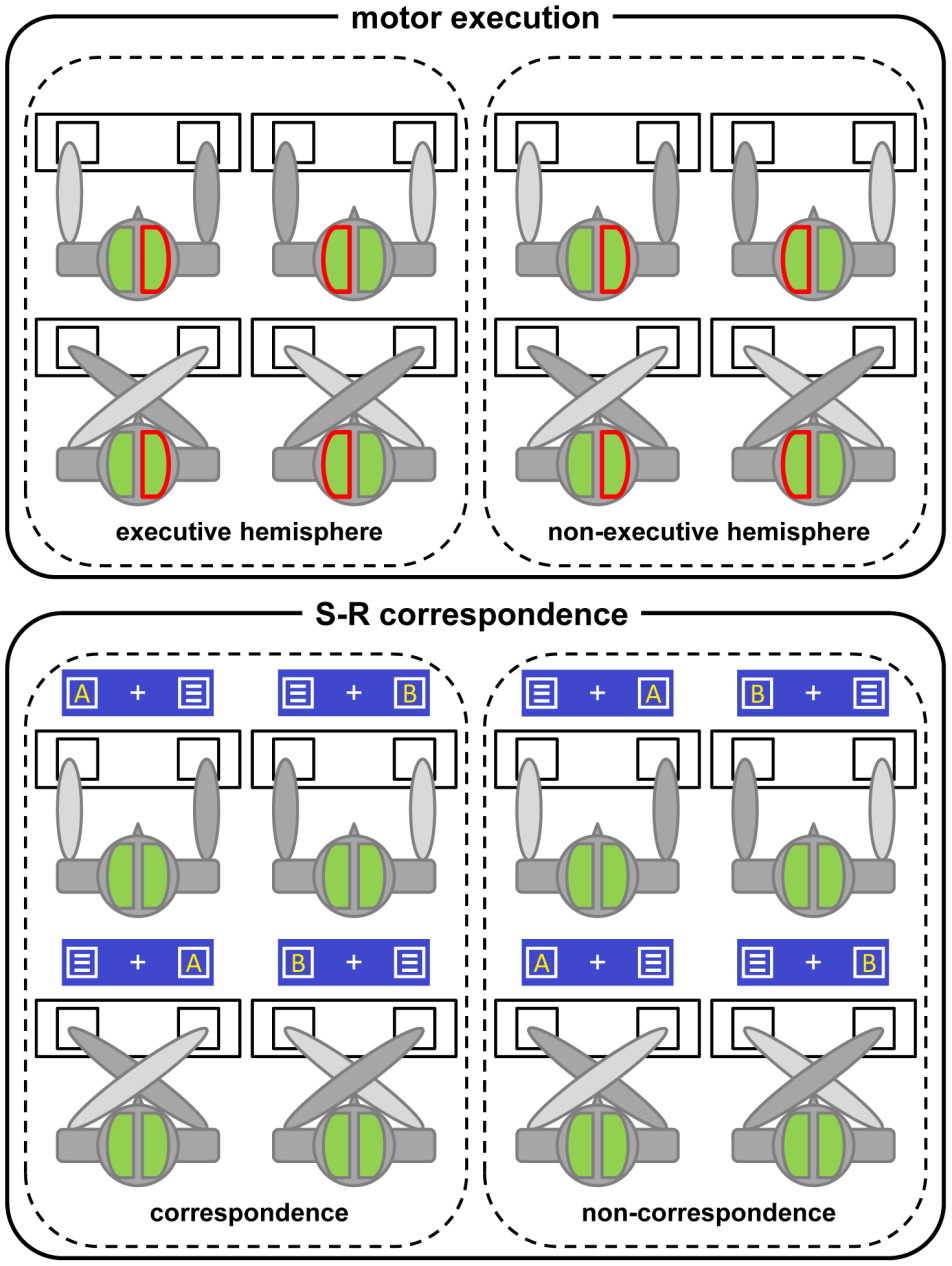
Visual illustration of experimental conditions/within-subject factors. The factor “motor execution” is depicted in the upper box. In the left section of that box, the executive hemisphere (the hemisphere responsible for the motor execution of the motor response) is marked red while in the right section of the box, non-executive hemisphere (the hemisphere not responsible for the motor execution of the motor response) is marked red. The factor “stimulus-response correspondence” is depicted in the lower box. Please note that there are parallel hands in the top rows of each of the four sub-boxes and crossed hands in the bottom rows. In a similar fashion, the left column of each of the four sub-boxes depicts left hand responses (the responding hand is indicated by light grey color), while the right column depicts right hand responses. In order to avoid explaining the obvious, we however refrained from explicitly depicting the conditions “used hand” (left anatomical hand vs. right anatomical hand) and “hand position” (parallel handy vs. crossed hands).

Behavioral data (RTs and error rates) were analyzed with the help of repeated-measures analyses of variance (ANOVA). Within-subject factors used were hand position (uncrossed vs. crossed), S-R correspondence (correspondent vs. non-correspondent) and used hand (left vs. right). The electrophysiological response-locked data was analyzed using three repeated-measures ANOVAs: Response-locked negative ERP peaks, the ERP peak-to-peak values and the evoked power extracted from two frequency bands (see TF decomposition) were separately analyzed, each using the within-subject factors hand position (uncrossed vs. crossed), S-R correspondence (correspondent vs. non-correspondent), used hand (left vs. right), and motor responsibility of the hemisphere (electrode above the hemisphere responsible for the motor execution of the response vs. electrode above the hemisphere irresponsible for the motor execution of the response). For an illustration of the within-subject factors, see Fig. 2. Greenhouse-Geisser-correction was used whenever necessary. All p-levels for post hoc t-tests were adjusted using Bonferroni correction. Effect sizes were given as the proportion of variance accounted for (η^2^). As a measure of variability, the standard error of the mean (SEM) together with the mean values was given. IBM SPSS statistics 20 was used for all statistical analyses.

## Results

### 3.1 Behavioral Data

Table 1 shows the mean percentage of hits and mean (RT) of correct responses (±SEM) in different conditions.

**Table 1:** Behavioral data.

	Mean percentage of hits		Mean RT in ms	
Conditions	Hits in %	SEM (hits)	RT in ms	SEM (RT)
right hand	91.17	0.896	410.00	7.100
left hand	91.00	1.105	420.00	8.290
parallel hands	93.08	0.926	408.00	7.368
crossed hands	89.09	1.090	422.00	7.934
S-R correspondence	95.60	0.719	395.74	7.183
S-R non-correspondence	86.57	1.330	435.00	8.240
correspondent parallel hands	95.92	0.680	391.00	7.300
non-correspondent parallel hands	90.24	1.370	426.19	7.800
correspondent crossed hands	95.28	0.850	399.74	7.400
non-correspondent crossed hands	82.90	1.727	444.61	9.160

#### 3.1.1 Percentage of correct responses

A repeated measures ANOVA for the percentage of hits revealed significant results for two main effects and two interactions: For hand position (F(1,24) = 29.06, p<.001, η^2^ = .548; parallel>crossed) and for S-R correspondence (F(1,24) = 79.14, p<.001, η^2^ = .76; correspondent>non-correspondent) (refer tab. 1).

For the interaction between hand position and S-R correspondence (F(1,24) = 11.91,p<.002, η^2^ = .33), post-hoc paired t-tests showed that the effect was due to a significant difference between hand positions in non-correspondent trials (t(1,24) = 4.49, p<.001) with parallel hands having a higher percentage of hits 90.42% (1.37) than crossed hands 82.90% (1.72). No such difference was found in correspondent trials (t(1,24) = −1.14, p<.262).

For the interaction between all used within-subject factors (hand position * correspondence * used hand; F(1,24) = 8.66,p<.007, η^2^ = .26) post-hoc repeated measures ANOVAs showed that the interaction between hand position and used hand was significant in correspondent trials (F(1,24) = 7.23, p<.013, η^2^ = 23.) but not in non-correspondent trials (F(1,24) = 2.10, p<.160, η^2^ = .081). Subsequent paired t-tests for the interaction in correspondent trials showed that the effect was due to a difference in the right hemifield/motor space: if the responding hand was placed on the right button, there was a significant difference (t(1,24) = 2.96, p<.007) due to the finding that parallel right-hand responses yielded a higher percentage of correct responses 96.80% (0.74) than crossed left-hand responses 94.64% (0.87). No such difference was found for the left hemifield/motor space (t(1,24) = −1.03, p<.313).

#### 3.1.2 Mean reaction times of correct responses

A repeated measures ANOVA for the mean RTs of correct responses revealed significant results for all three main effects: For hand position (F(1,24) = 24.00, p<.001, η^2^ = .500; parallel< crossed), for S-R correspondence (F(1,24) = 131.35, p<.001, η^2^ = .846; correspondent<non-correspondent), and for used hand (F(1,24) = 8.813, p<.007, η^2^ = .269; right<left) (refer tab. 1).

Additionally, there were two interactions: For the interaction between used hand and S-R correspondence (F(1,24) = 16.1,p<.001, η^2^ = .401) post-hoc paired t-tests showed that the effect was due to a significant difference between the used hands in non-correspondent trials (t(1,24) = 4.24, p<.001) with the left hand having a longer RT 443 ms (9.22) than the right hand 427 ms (7.61). No such difference was found in correspondent trials (t(1,24) = −1.145, p<.263).

For the interaction between all used within-subject factors (hand position * correspondence * used hand; F(1,24) = 10.01; p<.004; η^2^ = .294) post-hoc repeated measures ANOVAs showed that the interaction between hand position and used hand was significant in correspondent trails (F(1,24) = 7.15, p<.013, η^2^ = .230) but not in non-correspondent trials (F(1,24) = 3.82, p = .062, η^2^ = .137). Subsequent paired t-tests for the interaction in correspondent trials showed that the effect was due to a difference in the right hemifield/motor space: if the responding hand was placed on the right button, there was a significant difference between the left and right hand (t(1,24) = −3.17, p<.004) due to the finding that parallel right-hand responses yielded a shorter mean RT 385 ms (7.53) than crossed left-hand responses 401 ms (7.41). No such difference was found for the left hemifield/motor space (t(1,24) = −0.063, p<.950).

In short, the factors hand position and S-R correspondence had an influence on the percentage of hits as well as the mean RT of correct responses. An increase in the difficulty of each dimension (from parallel to crossed and/or from correspondent to non-correspondent) led to compromised performance (smaller percentage of hits/longer RTs). Additionally, the RTs of left hand responses were significantly slower than those of right hand responses. In both measures, the effect of the highest significant interaction could be attributed to differences observed in correspondent responses in the right hemifield/motor space (correspondent crossed hands left-hand responses vs. correspondent parallel hands right-hand responses). Also, left hand responses yield slightly worse results (significantly longer mean RTs and a non-significant tendency towards a lower percentage of hits).

### 3.2 Time Domain ERP Analysis and sLORETA

Prior to ICA, there were very few saccadic movements in the EEG raw data sets of all subjects. Therefore, we did not control for the number and direction of saccadic eye movements.

**Figure 3:**
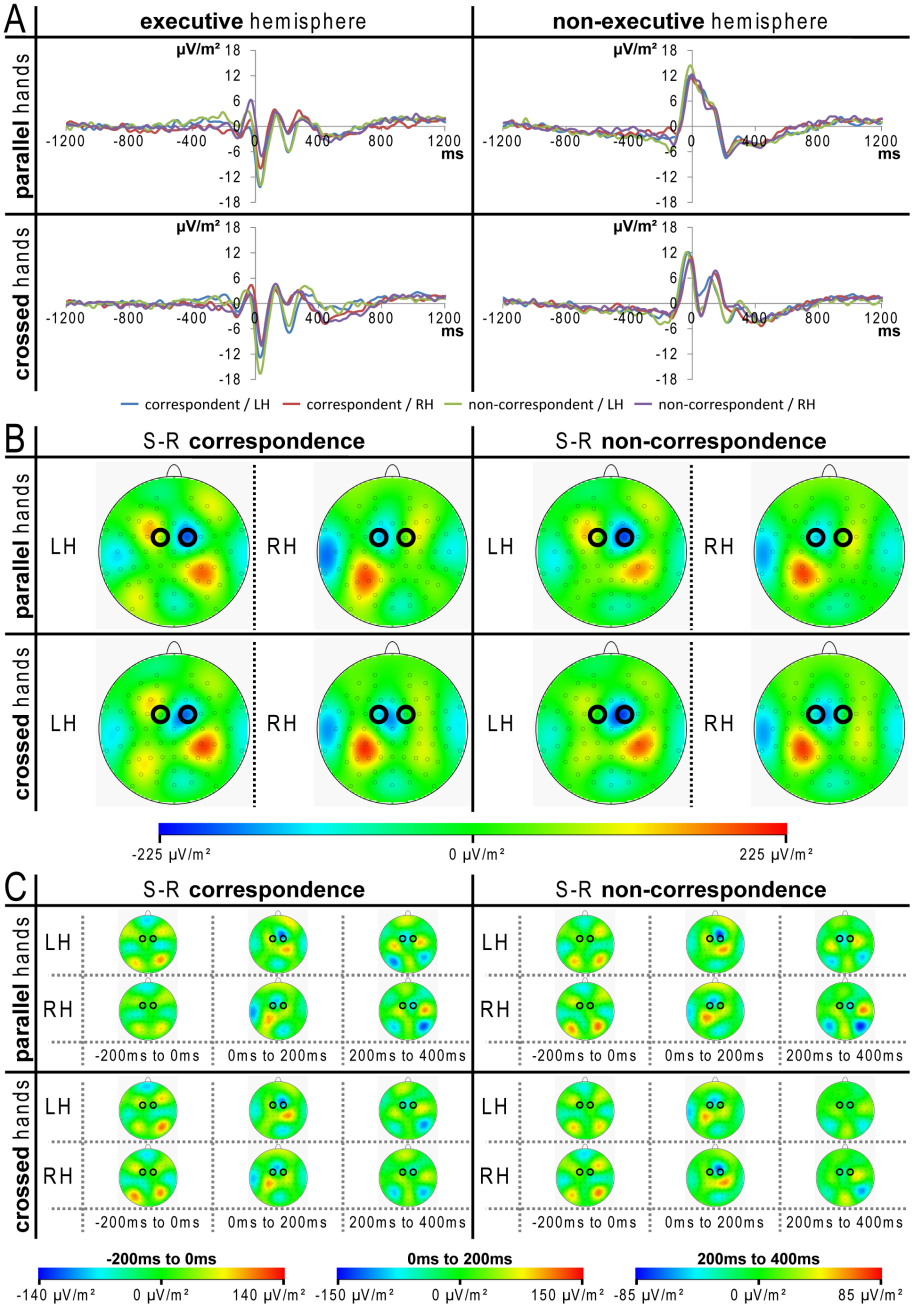
Response-locked ERPs and scalp topographies. Please note that all depicted results are based on CSD-transformed data. Hence, the units are given in µV/m^2^. A) Response-locked ERPs at electrodes FC1 and FC2. Based on the observed differences, the 16 different conditions were subdivided into four data sets/graphs according to hand position and motor execution (whether the hemisphere underneath the respective electrode was in charge of the motor execution of the response). Each graph contains four individual curves for all possible combinations of used hand and spatial S-R correspondence. As a result, each of the four graphs contains two ERP curves from FC1 and two ERP curves from FC2. Please note the post-response difference between the parallel and crossed hands ERP curves in the non-executive hemisphere (right column). Time point zero denotes the time point of response execution. B) Response-locked scalp topographies visualizing activity at the time point of the negative post-response peak used for data analyses. This time point was individually determined on the basis of the semiautomatic peak picking procedure applied to the data depicted in figure section A. Note that electrodes FC1 and FC2 (black circles) account best for the observed frontal amplitude changes. C) Averaged response-locked scalp topographies each comprising a 200 ms time interval covering the time span from −200 ms till 400 ms. The maps were obtained by averaging the signal of all electrodes over an interval of 200 ms (from −200 ms to 0 ms, from 0 ms to 200 ms and from 200 ms to 400 ms, respectively). Due to amplitude differences, different scale settings were used for the three epochs. Black circles were used to highlight the localization of electrodes FC1 and FC2 which were used for several statistical analyses. In this context it is important to note that due to the process of temporal averaging, the electrodes showing the most pronounced peaks/greatest changes in amplitude are not necessarily those in the center of topographically depicted negativations/positivations (compare figure section B).

The electrodes used for further analysis were chosen in a data-driven approach based on scalp topographies (see Fig. 3) and the minima/maxima of ERPs. Choosing the electrodes in the center of the topography, we aimed to maximize the obtained effects.

The results of the stimulus-locked data analysis can be found in the supporting information (Text S2).

#### 3.2.1 Response-locked ERP analysis

In Fig. 3 response-locked ERPs of the different conditions (hand position and correspondence) are plotted at electrodes FC1 and FC2. Fig. 3 also contains scalp topography maps based on data averaged over 200 ms time intervals as well as on the time point of the negative amplitude peak that we used in our analyses.

When the parallel hands (upper right graph) and the crossed hands (bottom right graph) ERP curves of the non-executive hemisphere are compared, a pronounced difference can be detected. Since we wanted to quantify response evaluation processes, we focused on the post-response negative peaks. However, due to the differences seen in the positive pre-response peak, the data for the repeated-measures ANOVA was quantified in two different ways (peak amplitudes and peak-to-peak values) in order to circumvent a possible bias induced by the quantification method. The results of the statistical analysis of the peak-to-peak values can be found in the supporting information (Text S3). Repeated-measures ANOVA of the response-locked negative post-response ERP peaks yielded significant main effects for hand position (parallel: −6.26 (1.30), crossed: −9.21 (1.31); F(1,24) = 12.80, p<.002, η^2^ = .348) and the hemispheric allocation of response motor execution (executive: −14.03 (1.57), non-executive: −1.43 (1.15); F(1,24) = 108.854, p<.001, η^2^ = .819). There were also two significant interactions: For the interaction between hand position and S-R correspondence (F(1,24) = 5.57, p<.027, η^2^ = .188), post-hoc paired t-tests revealed that it was due to a significant difference between correspondent and non-correspondent trials in the crossed hand condition (correspondent: −8.41, non-correspondent: −10.01; t(1,24) = 2.45, p<.011). There was no such effect in parallel hands (t(1,24) = -.54, p<.297). For the interaction between hand position and motor execution (F(1,24) = 25.15, p<.001, η^2^ = .512), post-hoc paired t-tests showed that the effect was due to a significant difference between parallel and crossed hands in the non-executive hemisphere (parallel: 1.34 (1.35), crossed: −4.21 (1.22); t(1,24) = 4.836, p<.001). The post-hoc test was non-significant in the executive hemisphere (t(1,24) = .446, p<.330).

In short, the results of these response-locked data analyses suggest that hand position as well as motor execution have an influence on neurophysiological processes after the execution of the response. Using the negative ERP peak values, S-R correspondence was also shown to influence activity levels as measured via EEG. The interaction between hand position and motor execution was driven by differences in the non-executive hemisphere where the parallel hands condition yielded a smaller activation than the crossed hands condition.

#### 3.2.2 sLORETA

**Figure 4:**
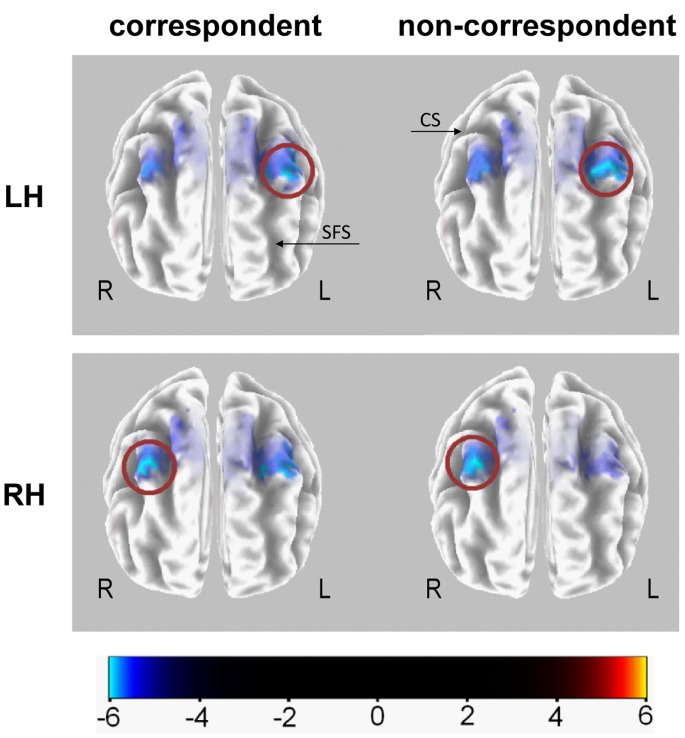
Source localization (response-locked). Top front view of the activation differences obtained via sLORETA analysis of the post-response ERPs. Crossed hands conditions were subtracted from parallel hands conditions. Only activation differences surpassing the significance threshold of p<.05 are depicted. As indicated by the blue color, the crossing of hands seems to have caused an increase in the activation in Brodman area 6/the middle frontal gyrus. Please note that the activation difference between parallel and crossed hands is bigger in the hemisphere which is not in charge of the motor response execution (red circles). This most probably depicts the post-response difference already observed in the RRPs shown in the right column of fig. 3a. The used hand (RH and LH) is denoted at the left side of the figure while the hemisphere (R and L) is indicated next to the respective hemispheres. To further help orientation, black arrows indicate the central sulcus (CS) and the superior frontal sulcus (SFS).

In Fig. 4, the significant activation differences between the conditions of parallel and crossed hand as revealed via sLORETA are mapped (*p*<.05, corrected for multiple comparisons). Within area BA6, the middle frontal gyrus/SMA showed an activation difference between hand positions. The sLORETA analysis corroborates the findings of the ERP analysis by showing that the crossing of hands led to a general bilateral activation increase and that this increase was more pronounced within the non-executive hemisphere.

#### 3.2.3 Time-frequency decomposition results

**Figure 5:**
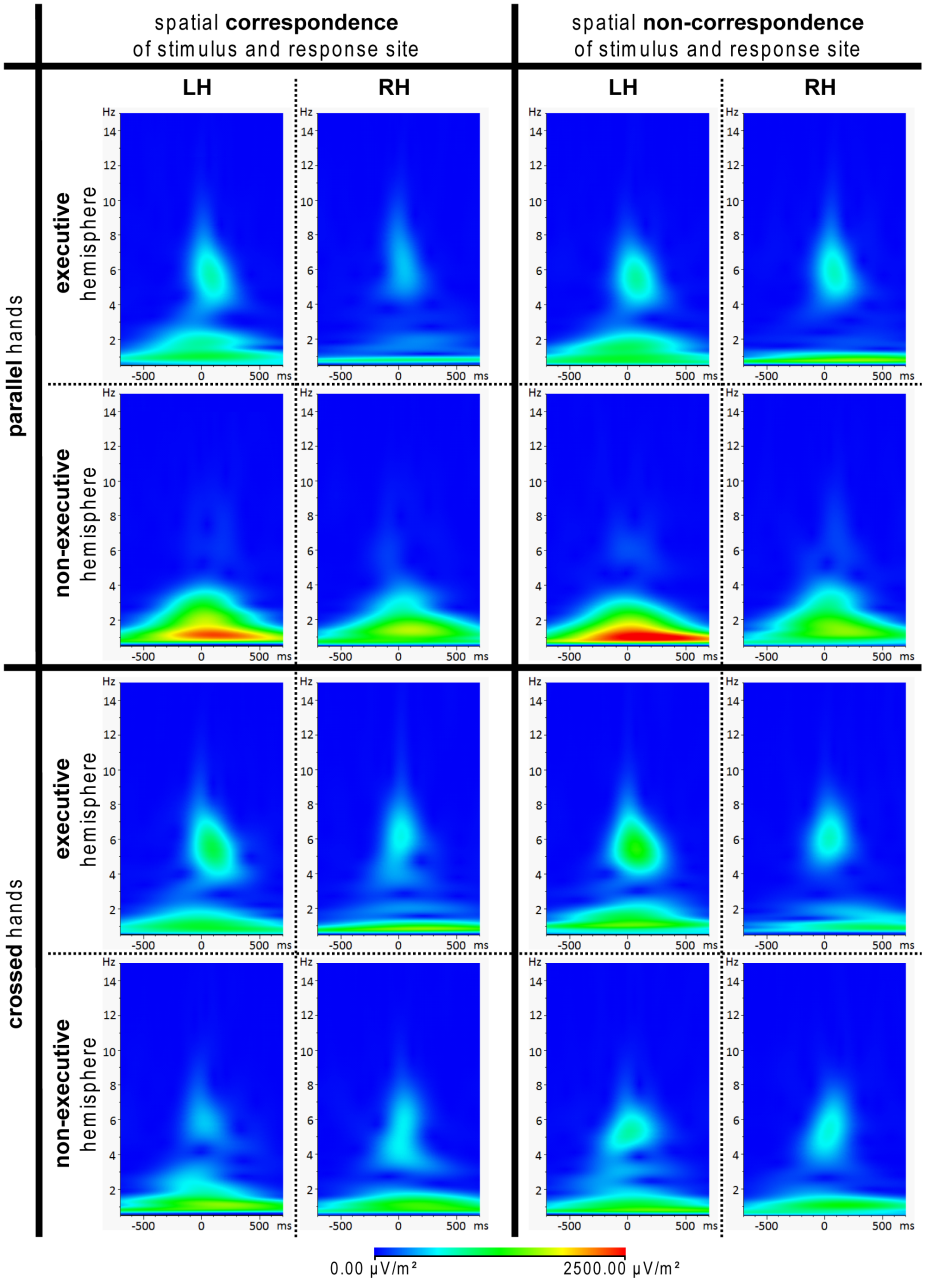
Response-locked TF decompositions/wavelets. Electrodes FC1 and FC2 were used to form response-locked TF decompositions for the 16 different conditions as defined via hand position, spatial correspondence, motor execution and used hand. As a result, FC1 was considered “non-executive” and FC2 was considered “executive” in left hand responses. In right-hand responses, this categorization was reversed. Please note the difference between the parallel and crossed hands TF plots in the non-executive hemisphere (2^nd^ vs. 4^th^ row).

In Fig. 5 response-locked wavelets of the different conditions (hand position and correspondence) are plotted at electrodes FC1 and FC2.

A repeated-measures ANOVA of the evoked power value peaks extracted from the time-frequency decomposition at 2.07 Hz showed a significant main effects of hand position (parallel: 3.02 (0.05), crossed: 2.88 (0.04); F(1,24) = 21.27, p<.001, η^2^ = .470). There were also three significant interactions: For the interaction between hand position and motor execution (F(1,24) = 14.10,p<.001, η^2^ = .370). Post-hoc paired t-tests revealed a significant difference between parallel and crossed hands in the non-executive hemisphere (parallel: 3.11 (0.07), crossed: 2.87 (0.06); t(1,24) = 5.667, p<.001). The post-hoc test was non-significant in the executive hemisphere (t(1,24) = 0.86, p<.398). For the interaction between hand position, used hand and motor execution (F(1,24) = 4.88,p<.037, η^2^ = .169) post-hoc repeated measures ANOVAs showed a significant interaction between motor execution and hand position in the left hand (F(1,24) = 22.91, p<.001, η^2^ = .488) but not in the right hand(F(1,24) = 1.68, p<.207, η^2^ = .066). Subsequent paired t-tests for the interaction in left hand trials showed that the effect was due to a difference in motor execution. In left-hand trials, there was a difference between hand positions in the non-executive hemisphere (parallel: 3.16 (0.08), crossed: 2.870 (0.07); t(1,24) = 4.91, p<.001). No such difference was found for the executive hemisphere (t(1,24) = −0.63, p<.531). For the interaction between hand position, S-R correspondence and motor execution (F(1,24) = 4.94,p<.036, η^2^ = .171), post-hoc repeated measures ANOVAs showed a significant interaction between hand position and S-R correspondence in the non-executive hemisphere (F(1,24) = 9.67, p<.005, η^2^ = .287) but not in the executive hemisphere (F(1,24) = 0.06, p<.809, η^2^ = .002). Subsequent paired t-tests for the interaction in the non-executive hemisphere showed that the effect was due to a difference in hand positions. In parallel hands, there was a difference between correspondent and non-correspondent trials (correspondent: 3.02 (0.08), non-correspondent: 3.203 (0.07); t(1,24) = −3.97, p<.001). No such effect was found in crossed hands (t(1,24) = 0.80, p<.430).

At 5.7 Hz, repeated-measures ANOVA of the evoked power peak values showed significant main effects for hand position (parallel: 2.79 (0.06), crossed: 2.95 (0.06); F(1,24) = 18.35, p<.001, η^2^ = .433), used hand (left hand: 2.94 µV/m^2^ (0.06), right hand: 2.80 (0.07); F(1,24) = 8.42, p<.008, η^2^ = .260), and motor execution (executive: 2.94 (0.07), non-executive: 2.80 (0.06); F(1,24) = 10.82 p<.003, η^2^ = .311). For the interaction between hand position and motor execution (F(1,24) = 10.67, p<.003, η^2^ = .308) post-hoc paired t-tests revealed that the interaction was due to a significant difference between the executive and the non-executive hemisphere in parallel hands (executive: 2.98 (0.07), non-executive: 2.92 (0.07); t(1,24) = 3.58, p<.001). There was no such difference in crossed hands (t(1,24) = 1.73, p<.087).

In short, most of the effects found were driven by differences in hand position (parallel hands) and motor execution (non-executive hemisphere). There were no significant differences among the wavelets of the executive hemisphere over the different conditions as defined by hand position and S-R correspondence. In parallel hands, the non-executive hemispheres differed both from the executive hemispheres and from each other (correspondent vs. non-correspondent trials). However, in crossed hands, there were no marked changes in activity across electrodes (executive vs. non-executive) or S-R correspondence (correspondent vs. non-correspondent). In addition to these findings, there was also a difference between the left and right hand in the delta band.

## Discussion

In the current study, we investigated how sensory and motor information are integrated in response evaluation processes. We hypothesized that for this purpose, efference motor copies and afferent proprioceptive information are most likely integrated within the SMA [5]–[7], [25]. To investigate this, hand position (posture) and S-R correspondence were varied using a Simon Task. The behavioral data as well as the stimulus-locked ERLs match previous findings obtained with crossed-hands versions of the Simon task (e.g. [61], [39], [71] see Text S1 and Text S2 for details). The drop in accuracy (number of hits) in crossed hands condition suggests that the postural change strips the subjects of some benefit available in normal (parallel) responses. According to our hypothesis, one of the reasons might be that the egocentric space is no longer aligned with representation of the responding hands. Even though spatial factors have been shown to be potential modulators of early stimulus-processing components [26], [32], [39], [61], the absence of effects (of hand position, used hand, motor execution of the hemisphere and spatial S-R correspondence) on electrodes PO7/PO8 suggests that early visual perception and attentional processing of the stimuli were neither influenced by the spatial proprioceptive information nor by the spatial S-R correspondence. Hence, the observed changes in response-locked ERPs are not likely to be due to attentional processing differences.

In contrast to this, response-locked ERP results match our predictions stating that changes in proprioceptive feedback lead to differences in post-response response monitoring processes. In this context, it needs to be emphasized that we would like to define the term “response monitoring” as comprising all aspects relevant to the evaluation of whether the response was executed as intended and whether it had the desired effects. Hence, the response monitoring processes discussed in this paper do not equal well-known concept of frontocentral (error) negativity as described by Falkenstein [72], Vidal et al. [73], Ullsperger and von Cramon [74], Beste et al. [40], [66] and many others. These differences found in this study were most evident in the hemisphere that is not in charge of the motor execution of the response. In the TF decomposition, the modulatory effect of proprioception was reflected in the delta and theta frequency bands. More precisely, activation differences between the hemispheres seemed to vanish in crossed hands as the non-executive hemisphere conformed to the activation pattern of the executive hemisphere (see Fig. 5). Therefore, it can be stated that the external spatial location of the hands coded by proprioceptive information probably has an effect on different processes as reflected by different frequency bands [17]. In contrast to this, the effect of efference motor copies/motor response execution seemed to be more confined to the theta frequency band which nicely matches the observations of Therrien et al. [75] who demonstrated the link between efference motor copies and theta oscillations. Also, the theta frequency band has been suggested to play an important role in response monitoring processes [17], [47], [51]–[52] providing further support that the post-response modulation of the theta band might be the measure of a general, central executive-guided response evaluation process incorporating different kinds of information.

The sLORETA analyses of the response-locked ERP data strongly suggests that these differences in modulation of post-response ERPs were due to activity changes in Brodman area 6 (middle frontal gyrus), which has repeatedly been linked to response and goal-selection conflicts [76]–[78]. This finding is also corroborated by the involvement of SMAs which are known to process both efference copies of motor responses [4]–[9], [25] and afferent proprioceptive information [5]–[7], [25].

Our findings have several implications: First of all, proprioceptive information seems to influence response monitoring processes. Second, the finding that crossing hands changes behavioral and electrophysiological measures supports the conclusion that the effector’s position in space is coded in an external reference frame. This implies that in the case of proprioceptive information, the originally somatotopic input undergoes a remapping process during which it is transformed into external spatial coordinates [26], [32], [36]–[37]. Third, efference motor copies do not seem to be subject to major spatial remapping since this would not comply with the finding of increased bilateral activation in crossed hands. Yet, due to findings on slight ipsilateral hemispheric activation during unimanual movements [5] we cannot fully rule out the possibility of minor efference copy remapping. Fourth, the general activation increase evoked by the crossed hands posture could be explained either by a processing conflict between the hemispheres or by an increased effort to integrate the dispersed pieces of information (compare [26], [32]). If the latter were to hold true, we could conclude that within the SMA, both anatomical (efference motor copies) and external spatial information are integrated in order to obtain a stable combined representation of internal and external events for the purpose of response monitoring and subsequent behavioral modifications (if necessary).

**Figure 6:**
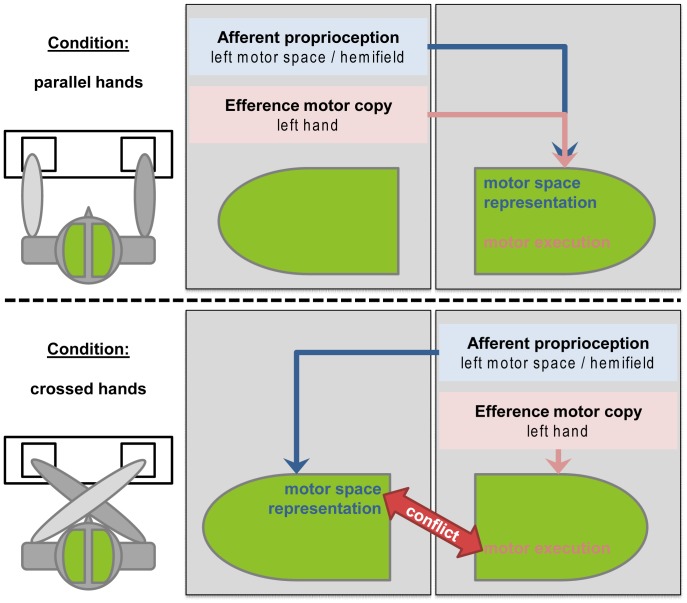
Results-based theoretical model. Given that the execution of the motor response and the spatial representation of the motor space are immutably locked to the two hemispheres of the brain [7], [25], [30], crossing hands (entering the "foreign” motor space) may impose a conflict. The consequences of an independent allocation of efferent and afferent information illustrated for left-hand responses. In crossed hands only, one hemisphere is executing the motor response while the response itself physically takes place in the motor space represented in the opposite hemisphere of the brain. For right hand responses, the allocation is mirror-inverted.

Based on these findings, we therefore propose a model based on external events and their hemispheric allocation in order to offer an explanation for the observed data pattern. In most studies, external references like lateral proximity of stimulus and effector (S-R correspondence) are used to categorize conditions in the Simon Task [61]. However, this allocentric external approach does not take into account how variations in these dimensions affect the way we process the involved information. For example, crossing of hands changes their spatial proximity to the stimulus and their position in space, but it neither changes which hemisphere processes the stimulus, nor which hemisphere is needed for the motor execution of the response. In contrast, changing the location of a stimulus changes the hemisphere in which it is first visually processed and the proximity to the responding hand, but it does neither change the spatial position of the responding hand nor which hemisphere is in charge of the motor execution of the response. The spatial representation of the two sides of the body is changed by neither of the two manipulations, but the allocation of stimulus processing, limbs and response buttons can be varied within the egocentric visual hemifields. It is therefore important to consider the effects with respect to the division of labor between the hemispheres and how this information is integrated across hemispheres. The consequences of this approach are depicted in Fig. 6. Our model illustrates the main difference between the two hand positions: in crossed hands only, one hemisphere is executing the motor response while the response itself physically takes place in the motor space represented in the opposite hemisphere of the brain.

Since proprioceptive information of resting limbs seems to be a rather tonic input [23], the consequent interhemispheric information processing might also put an additional strain on earlier processing steps and thus possibly account for a decrease in task performance. Even though not depicted in Fig. 6, the necessity of interhemispheric information transfer also provides an explanation for the effects of the spatial (non-)correspondence of stimulus and reaction sites: Interhemispheric transfer also needs to take place in situations in which the initial visual processing and the motor execution of the response are carried out in different hemispheres [79]. However, it is important to point out that even though the S-R correspondence biases the response and thus produces behavioral differences [61], it ought to be rather irrelevant to the action goal representation (the only relevant stimulus information is its categorization: letter A or B?). In other words, information on stimulus lateralization is not mandatory for a proper response evaluation in the Simon Task. This assumption matches the finding that S-R correspondence seemed to have a rather small impact on response evaluation processes.

Yet still, there are a few limitations to our data. In some of the analyses, we found differences between the used hands. Since all of our subjects were right-handed and always placed the left arm above the right arm when crossing hands, we cannot find out whether the differences between left and right hand responses were produced by handedness, the crossed hands posture or an interaction of both factors. Another possible explanation is that for right-handed subjects it might more difficult to remap their dominant hemifield in the crossed hands condition [80]. The faster sign was presented in all trials with an RT longer than 500 ms. This could potentially induce systematic differences since the RTs of the conditions were not equal (compare tab. 1). However, we chose not to analyze this potential bias because non-correspondent crossed hands had an average RT of 444 ms (SEM = 9.160) which suggests that even in the slowest condition, the majority of trials was below the 500 ms criterion. Also, we expect visual disturbances (that do not indicate an error) to have a rather minor effect on the response evaluation processes. Last but not least, one might argue that the visual perception of one’s hands might have intermingled the effects of proprioception resulting in visuoproprioceptive integration [4]. However, the head support fixating the subjects’ heads in front of the monitor made it very hard to visually perceive one’s hands so that only 4 out of 25 subjects reported being able to see their hands during the task.

### Conclusions

Summing up our findings, it can be stated that motor response evaluation requires information about different aspects of the respective response. Even though both pieces of information seem to be at least partly processed within the SMA/middle frontal gyrus, efference motor copies and afferent proprioceptive information are clearly based on discrete sources and exert their effects differently. Efference motor copies seem to be largely retained in the hemispheres in which they were generated and efference copy based information about the motor execution of a response has a rather specific effect that is most prominent in the theta frequency band. In contrast to this, afferent proprioceptive input is used to determine the effector’s spatial location in space which seems to be allocated in an external reference frame. Also, afferent proprioceptive information has a rather broad influence on response evaluation (as reflected by changes in different frequency bands).

Based on our results, we conclude that a crossed hands posture induces a bilateral allocation of these inputs, changing the patterns of neuronal activation and augmenting overall activity. This allows for to the conclusion that within the middle frontal gyrus and the SMA, cross-modal integration takes place for the purpose of response evaluation. Also, the dissociation of spatial and motor information illustrates the modularity and flexibility of response evaluation components: By simply asking our subject cross their hands so that they enter the “foreign” hemifield, we were able to allocate parts of the response evaluation within the hemisphere that would otherwise not have participated in this process. We were thus able to demonstrate that the allocation of our actions in egocentric space plays a considerable role in response monitoring processes.

## Supporting Information

Text S1 **Vincentizing procedure.**
(PDF)

Text S2 **Stimulus-locked ERLs.**
(PDF)

Text S3 **Analysis of response-locked peak-to-peak ERP data.**
(PDF)
