# Supplementary material for: Differential Effects of Motor Efference Copies and Proprioceptive Information on Response Evaluation Processes
Source: PLoS One. 2013 Apr 26;8(4):e62335. doi: 10.1371/journal.pone.0062335 (PMC3637248; doi:10.1371/journal.pone.0062335)
Supplement: Text S1 — Vincentizing procedure. (PDF) [file pone.0062335.s001.pdf]

<u>Supplement 1</u>

Vincentizing procedure

In order to make sure that the model introduced in our discussion is based on data comparable to the results of previous studies employing the Simon Task, we performed a vincentizing procedure (Leuthold, 2011; Proctor, 2011; Simon, 1990; Wascher et al, 2001; Wiegand and Wascher, 2005). The mean results of the application of the vincentizing procedure to the individual RT data are plotted below.

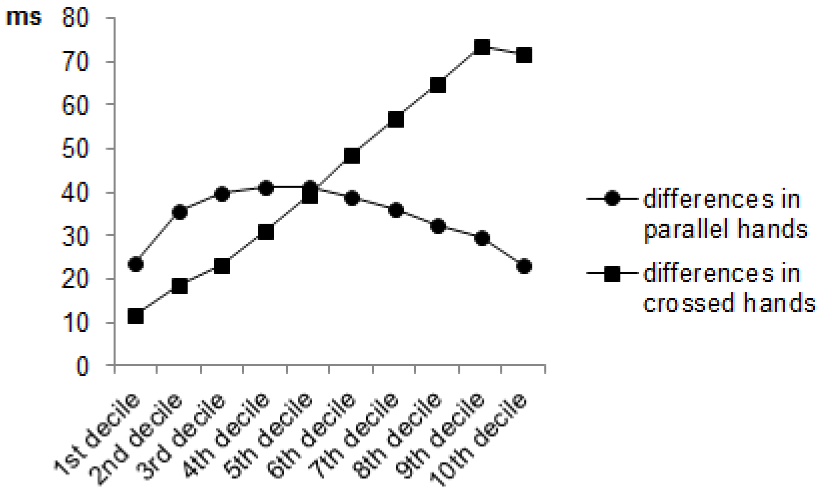

Figure S-1

The plot denotes differences between crossed and parallel hands in the mean RTs of each decile (for the purpose of forming deciles, individual RT data was sorted in an ascending order). To obtain difference values, the mean RT of spatially correspondent trials was subtracted from that of spatially non-correspondent trials (see Wiegand & Wascher, 2007 or Ratcliff, 1979 for further information). The different slopes of the two resulting curves illustrate that an automatic activation of the corresponding response resulting in a facilitation of this response only occurs under specific circumstances (including the “natural” parallel hand position) and are in line with the effects already reported in prior studies of the Simon

Task (Leuthold, 2011; Proctor, 2011; Simon, 1990; Wascher et al, 2001; Wiegand and Wascher, 2005, 2007). The stimulus –locked ERLs also match previous findings in this field (e.g. Wiegand & Wascher, 2005).

Leuthold H (2011) The Simon effect in cognitive electrophysiology: a short review. Acta Psychol 136: 203-211.

Proctor RW (2011) Playing the Simon game: use of the Simon task for investigating human information processing. Acta Psychol (Amst) 136(2): 182-8.

Ratcliff R (1979) Group reaction time distributions and an analysis of distribution statistics. Psychol Bull 86(3): 446–461.

Simon J R (1990) The effects of an irrelevant directional cue on human information processing. In: Stimulus-response correspondence: An integrated perspective (Proctor RW, Reeve TG (Eds.)); pp. 31−86. Amsterdam: Elsevier.

Wascher E, Schatz U, Kuder T, Verleger R (2001) Validity and boundary conditions of automatic response activation in the Simon task. J Exp Psychol Hum Percept Perform 27(3): 731-51.

Wiegand K, Wascher E (2005) Dynamic aspects of stimulus-response correspondence: evidence for two mechanisms involved in the Simon effect. J Exp Psychol Hum Percept Perform 31(3):453-464.

Wiegand K. & Wascher E. (2007) Response coding in the Simon Task. Psychol Res 71: 401–410.
