# Supplementary material for: Differential Effects of Motor Efference Copies and Proprioceptive Information on Response Evaluation Processes
Source: PLoS One. 2013 Apr 26;8(4):e62335. doi: 10.1371/journal.pone.0062335 (PMC3637248; doi:10.1371/journal.pone.0062335)
Supplement: Text S2 — Stimulus-locked ERLs. (PDF) [file pone.0062335.s002.pdf]

Supplement 2

Stimulus-locked ERLs: Methods

For the analysis of stimulus-locked event-related potentials (ERPs), segments were formed for the different conditions. Since the main focus of this study lies on frontal response-locked processes, the main purpose of the calculation of stimulus-locked ERLs is to validate our findings by showing that they resemble findings made in previous studies of the Simon task (Leuthold 2011; Wascher et al. 2001). These epochs started 200ms before the onset of the stimulus presentation (which was set to time point zero) and ended 1200ms after the stimulus onset, resulting in an epoch length of 1400ms. Only trials that had been correctly answered within the first 1500ms after the onset of the stimulus presentation were included. An automated artifact rejection procedure was run using a maximum voltage step of more than 50 µV/ms, a maximal value difference of 200 µV in a 200 ms interval, or activity below 0.5 µV as rejection criteria. To re-reference the data, a current source density (CSD) transformation was applied. The CSD transformation eliminates the reference potential (see: Nunez et al. 1997; Perrin et al. 1989; Pernier et al. 1988). For the time-domain analysis, a baseline correction was applied from -200ms to 0ms (i.e., stimulus onset) to eliminate background activity. The epochs of the different conditions were then averaged. Using the same conditions (correspondent & parallel hands / non-correspondent & parallel hands / correspondent & crossed hands / non-correspondent & crossed hands) of the two hands, event-related lateralizations (ERLs) were calculated for PO7/PO8, C3/C4, and FC1/FC2. As a result of combining the different used hand conditions, each of the obtained ERLs is a combination of left and right hand trials. A semiautomatic peak detection was run to identify global maxima (for the correspondent parallel hands and non-correspondent crossed hands conditions) and minima (for the non-correspondent parallel hands and correspondent crossed hands conditions) in a time frame of 150-300ms after the onset of stimulus presentation. Since

this process generated ERLs with a different polarity from data with originally identical polarity the ERL values were subsequently corrected for polarity.

For the peaks extracted from the stimulus-locked ERLs, hand position (uncrossed vs. crossed) and S-R correspondence (correspondent vs. non-correspondent) were used as within-subject factors.

Stimulus-locked ERLs: Results

In Fig. S-2 the stimulus-locked ERLs for FC1/FC2, C3/C4, and PO7/PO8 are depicted together with time-averaged scalp topography maps covering the range of 0 ms till 400 ms. Due to the prior CSD transformation, units for amplitudes are given in $\mu V/m^2$.

Due to the extensive statistical analysis of response-locked data described below, only the ERL peaks for electrodes PO7/PO8 were included in the stimulus-locked statistical analysis. The repeated-measures ANOVA of the stimulus-locked ERL peaks for electrodes PO7/PO8 as depicted in Fig. 2 revealed a significant effect of the interaction between hand position and S-R correspondence ($F(1,24)=90.11$, $p<.001$, $\eta^2=.790$). However, after correcting for ERL polarity, no significant effects for any of the within-subject factors remained (all $F(1,24)\leq 3.5$, $p\geq .077$, $\eta^2\leq .125$). Thus, the results suggest that the early visual processing of the stimuli as measured via stimulus-locked ERL peaks were neither influenced by hand position nor by S-R correspondence.

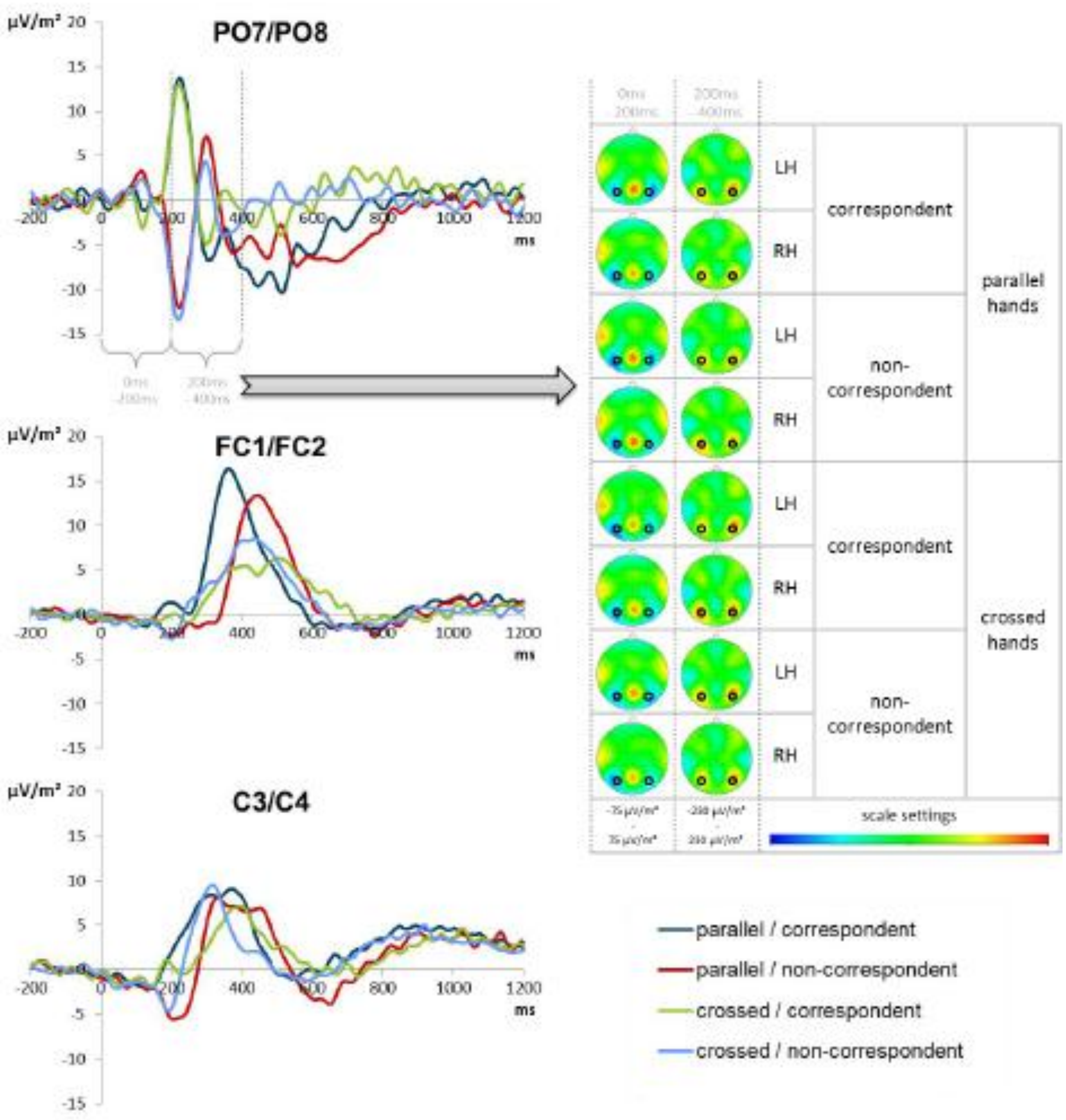

Figure S-2

Figure: Stimulus-locked ERLs and scalp topographies

Stimulus-locked ERLs at electrodes FC1/FC2, C3/C4 and PO7/PO8 are shown in the three line graphs on the left side of the figure. Time point zero denotes the time point of stimulus presentation. The different conditions depicted in the ERL graphs are explained in the bottom right corner. The table on the right hand side comprises ERP-based scalp topography maps. These maps were obtained by averaging the signal of all electrodes over the course of 200 ms

(from 0ms to 200ms and from 200ms to 400ms, respectively). Please note that due to amplitude differences, different scale settings had to be used for the two epochs. In addition to this epoch information, dotted lines in the PO7/8 ERL plot indicate the boundaries of the time spans used and black circles point out the localization of electrodes PO7 and PO8 which were used for statistical analyses. The peaks seen in the ERLs of electrodes FC1/FC2 and C3/C4 at about 400ms most likely depict lateralized readiness potentials / response-related neuronal activity as described by Stürmer et al. (2002) or Leuthold (2011).

Leuthold H (2011) The Simon effect in cognitive electrophysiology: a short review. Acta Psychol 136: 203-211.

Wascher E, Schatz U, Kuder T, Verleger R (2001) Validity and boundary conditions of automatic response activation in the Simon task. J Exp Psychol Hum Percept Perform 27(3): 731-751.

Nunez PL, Srinivasan R, Westdorp AF, Wijesinghe, RS, Tucker DM, et al. (1997) EEG coherency. I: Statistics, reference electrode, volume conduction, Laplacians, cortical imaging, and interpretation at multiple scales. Electroencephalogr Clin Neurophysiol 103: 499-515.

Perrin F, Pernier J, Bertrand O, Echallier JF (1989) Spherical splines for scalp potential and current density mapping. Electroencephalogr Clin Neurophysiol72(2): 184-187.

Pernier J, Perrin F, Bertrand O (1988) Scalp current density fields: concept and properties. Electroencephalogr Clin Neurophysiol 69(4):385-389.

Stürmer B, Leuthold H, Soetens E, Schröter H, Sommer W (2002) Control over location-based priming in the Simon task: Behavioral and electrophysiological evidence. J Exp Psychol Hum Percept Perform 28: 1345-1363.
