# Supplementary material for: Differential Effects of Motor Efference Copies and Proprioceptive Information on Response Evaluation Processes
Source: PLoS One. 2013 Apr 26;8(4):e62335. doi: 10.1371/journal.pone.0062335 (PMC3637248; doi:10.1371/journal.pone.0062335)
Supplement: Text S3 — Analysis of response-locked peak-to-peak ERP data. (PDF) [file pone.0062335.s003.pdf]

Supplement 3

Analysis of response-locked peak-to-peak ERP data: Results

Repeated-measures ANOVA of the response-locked peak-to-peak values yielded significant main effects for hand position (parallel: 16.61 (1.40), while crossed: 19.37 (1.812); $F(1,24)=8.95$, $p<.006$, $\eta^2=.272$), S-R correspondence (correspondent: 17.05 (1.55), while non-correspondent: 18.93 (1.67); $F(1,24)=4.57$, $p<.043$, $\eta^2=.160$), and motor execution (executive: 20.63 (1.94), while non-executive: 15.34 (1.38); $F(1,24)=16.36$, $p<.001$, $\eta^2=.405$). Additionally, there was one significant interaction between hand position and motor execution ($F(1,24)=7.55$, $p<.011$, $\eta^2=.239$). Post-hoc paired t-tests revealed that the interaction was due to a significant difference between parallel and crossed hands in the non-executive hemisphere (parallel: 13.01 (1.43), while crossed: 17.67 (1.69); $t(1,24)=-3.10$, $p<.003$). There was no such effect in the executive hemisphere ($t(1,24)=-1.33$, $p<.098$).
